# Clinical and psychological features of children and adolescents diagnosed with avoidant/restrictive food intake disorder in a pediatric tertiary care eating disorder program: a descriptive study

**DOI:** 10.1186/s40337-018-0193-3

**Published:** 2018-04-27

**Authors:** Megan Cooney, Melissa Lieberman, Tim Guimond, Debra K. Katzman

**Affiliations:** 1 Division of Adolescent Medicine, Department of Paediatrics, Hospital for Sick Children and University of Toronto, 555 University Avenue, Toronto, ON M5G 1X8 Canada; 2 Department of Psychology, Eating Disorders Program, Hospital for Sick Children, 555 University Ave, Toronto, ON M5G 1X8 Canada; 3 Mental Health Service, St. Michael’s Hospital and University of Toronto, 30 Bond Street, Toronto, ON M5B 1W8 Canada

**Keywords:** Eating disorder, Avoidant/restrictive food intake disorder, Weight loss, Children, Adolescents

## Abstract

**Background:**

Avoidant/Restrictive Food Intake Disorder (ARFID) is an eating disorder first described in the *Diagnostic and Statistical Manual of Mental Disorders, 5th edition* (DSM-5) [American Psychiatric Association, Diagnostic and statistical manual of mental disorders, 2013]. Patients with ARFID do not fear gaining weight or have body image distortions. ARFID involves a persistent disturbance in feeding and eating that results in an inability to meet nutritional and/or energy needs with one of the following: weight loss or failure to achieve appropriate weight gain, nutritional deficiency, dependence on enteral feeding or nutritional supplements and significant interference with psychosocial functioning. To date, studies on patients with ARFID have retrospectively applied the DSM-5 diagnostic criteria for ARFID to reclassify patients diagnosed with DSM-IV eating disorders.

**Methods:**

A descriptive retrospective chart review was completed on patients less than 18-years diagnosed with ARFID after a comprehensive eating disorder assessment between May 2013 and March 2016. The data collected included demographics, anthropometrics, historical information, clinical features, co-morbid diagnoses, need for inpatient hospitalization and psychometric measures.

**Results:**

Three hundred and sixty-nine patients were assessed for an eating disorder between May 2013 and March 2016. Of these, 31 (8.4%) received a DSM-5 diagnosis of ARFID. A full chart review was performed on 28 (90.3%) patients. Weight loss or failure to achieve appropriate weight gain was the reason for diagnosis in 96.4% (27/28). All of our patients had 2 or more physical symptoms at the time of diagnosis and 16 (57.1%) had a co-morbid psychiatric disorder. Twenty (71.4%) reported a specific trigger for their eating disturbance. Admission for inpatient hospitalization occurred in 57.1% (16/28) of patients. Thirteen (46.4%) patients had been previously assessed by another specialist for their eating disturbance. None of the patients had elevated scores on commonly used psychometric tests used to assess eating disorders.

**Conclusion:**

This is the first study to retrospectively determine the incidence of ARFID in children and adolescents using the DSM-5 diagnostic criteria at assessment. The clinical presentation of patients with ARFID is complex with multiple physical symptoms and comorbid psychiatric disorders. Commonly used pediatric eating disorder psychometric measures are not specific for making a diagnosis of ARFID, and may not be sensitive as assessment tools.

## Background

Avoidant/Restrictive Food Intake Disorder, also known as ARFID, is an eating disorder that was first described in the 5th edition of the *Diagnostic and Statistical Manual of Mental Disorders* (DSM-5). ARFID involves a persistent disturbance in feeding and eating that results in an inability to meet nutritional and/or energy needs and leads to at least one of the following: weight loss or failure to achieve appropriate weight gain; nutritional deficiency; dependence on enteral feeding or nutritional supplements; or significant interference with psychosocial functioning [1]. ARFID replaces and expands on the DSM-IV diagnosis of feeding disorder of infancy and early childhood [2]. Unlike patients with anorexia nervosa (AN), those with ARFID do not fear gaining weight and are not preoccupied with their body weight, shape or size. As it is presently described, the broad clinical features of the ARFID classification encompasses a heterogeneous patient population.

Previous studies examining the incidence and clinical characteristics of children and adolescents with ARFID in pediatric tertiary care eating disorder programs have found the incidence to be between 5 and 14% [3, 4]. These studies found that patients with ARFID were more likely to be younger and male, have a longer duration of illness, and present more often before the age of 12 compared to patients with AN or bulimia nervosa (BN). Another study found the prevalence of ARFID in an eating disorder day hospital to be 22.5% [5]. However, these studies retrospectively applied the DSM-5 diagnostic criteria for ARFID to reclassify children and adolescents diagnosed with DSM-IV eating disorders. An Australian population-based survey assessed for diagnostic features of DSM-5 eating disorders in individuals over age 15 years and found the 3-month prevalence of ARFID and AN to be 0.3% and 0.4%, respectively [6]. However, there is no literature describing the incidence and clinical presentation of children and adolescents with ARFID prospectively following a comprehensive eating disorder^1^ assessment in a tertiary care pediatric eating disorder program.

In addition, very little information is known about how patients with a clinical diagnosis of ARFID respond on commonly used eating disorder psychometric measures. A 4-year retrospective chart review of children and adolescents admitted to an eating disorder day program used the Children’s Eating Attitudes Test (ChEAT) to assess eating disorder symptoms. Patients meeting criteria for ARFID had significantly lower total scores on this measure relative to patients meeting criteria for AN and BN, indicating fewer classic eating disorder symptoms [6]. They had a higher comorbidity of anxiety disorders, pervasive developmental disorders and learning disorders and a lower comorbidity of depressed mood, as assessed using the Children’s Depression Inventory (CDI), Revised Children’s Manifest Anxiety Scale and The Child Behavior Checklist, compared to patients with other eating disorders. This chart review also involved patients that were retrospectively reclassified using the DSM-5 diagnostic criteria. There is no literature describing psychometric measures for children and adolescents newly diagnosed with ARFID during a comprehensive assessment.

The objective of this study was to determine the incidence of ARFID using the DSM-5 diagnostic criteria at assessment in a pediatric tertiary care eating disorder program and describe the clinical and psychological characteristics of children and adolescents with ARFID.

## Methods

A retrospective chart review was completed. All patients under the age of 18 years who received a diagnosis of ARFID after a comprehensive eating disorder assessment in a tertiary care pediatric hospital between May 2013 and April 2016 were included in this study. The comprehensive assessment consisted of a diagnostic evaluation with the patient and with family members by either a trained psychiatrist or psychologist using the DSM-5 criteria, medical assessment by an adolescent medicine specialist or nurse practitioner, a nutritional assessment by a dietician with experience in eating disorders and a battery of psychometric measures.

### Demographics, historical and clinical features

Data collected comprised of information that existed in the patient’s medical record at the time of the assessment. Data included age, gender and ethnicity; vital signs including heart rate, blood pressure and temperature; EKG findings, including a calculated QTc interval; blood work including complete blood count, potassium, phosphate, magnesium, calcium, sodium, glucose; duration of illness (defined as the reported outset of nutritional restriction up until the date of the assessment); highest and lowest reported weights in the last year; daily caloric intake (24-h dietary recall) at time of assessment by the dietician; menstrual and pubertal status; referral source; and the presence of coexisting medical conditions. Additional information collected included food allergies; history of a choking episode; history of food avoidance or food refusal; purging; excessive exercise, defined as exercise for 7 or more hours per week or any regular exercise accompanied by moderate to severe distress when the patient cannot exercise [6]; enteral supplement use; associated symptoms such as dysphagia, abdominal pain, fear of vomiting, generalized anxiety with eating, early satiety, nausea, sensory issues related to food texture and smell; and a history of being a picky eater. Finally, reported presence of past history or present co-morbid psychiatric diagnosis, family history of an eating disorder or other psychiatric diagnosis were also collected.

### Anthropometrics

Weight in a gown and height were measured by nursing staff. Body mass index (BMI) was calculated (kg/m2) and BMI percentile was determined using WHO growth charts. Target goal weight (TGW) was determined by dieticians and physicians using the patient’s pre-morbid growth trajectory in height and weight and pubertal stage [7]. Percentage of TGW and percentage of body weight lost, defined as current weight divided by weight before the onset of the eating disturbance were determined.

### Psychometric measures

The eating disorder assessment at SickKids includes a psychometric battery of tests: Children’s Depression Inventory (CDI), the Multidimensional Anxiety Scale for Children (MASC), the Eating Disorder Examination Questionnaire (EDE- Q), the Children’s Eating Attitude Test (ChEAT), the Eating Disorder Inventory for Children (EDI-C) and the Eating Disorder Inventory - 3 (EDI-3) were collected. Of note, in the spring of 2015 the Department of Psychology at SickKids started using the CDI 2 instead of the CDI and the MASC 2 instead of the MASC. Although data from the different versions of the CDI and MASC cannot be analyzed together, data from both measures were analyzed separately and compared to look for overall markers of depression and anxiety respectively, as well as overall trends on the specific subscales, most of which are similar between the two versions of the measures.

The CDI is a 27-item self-report measure that evaluates depression in children and adolescents ages 7–17 years [8]. It consists of 5 subscales: Negative mood, Interpersonal problems, Ineffectiveness, Anhedonia, and Negative Self-esteem. It also provides a total score. The CDI 2 is a revision of the CDI [9]. It contains 2 scales: Emotional problems and Functional problems and 4 subscales: Negative mood, Negative self-esteem, Ineffectiveness, and Interpersonal problems. It also provides a total score. To allow for comparisons between versions, T scores of 65 and higher were considered elevated.

The MASC is a 39-item self-reported measure used to assess anxiety in ages 8–19 years [10]. It includes 4 scales: Physical symptoms, Harm avoidance, Social anxiety, and Separation/Panic. It also measures total anxiety. The MASC 2 is a revision of the MASC and contains 50 items across 6 scales: Separation anxiety/ Phobias, GAD index, Social anxiety, Obsessions and compulsions, Physical symptoms and Harm avoidance. It also provides a total score [11]. T scores of 65 and higher were considered elevated.

The EDI-3 is a self-report measure of psychological traits in individuals with eating disorders ages 13–53 years [12]. It has 91 items organized in 12 primary scales, in which 3 are eating disorder specific: Drive for thinness, Bulimia, and Body dissatisfaction. Nine are general psychological scales: Low self-esteem, Personal alienation, Interpersonal insecurity, Interpersonal alienation, Interoceptive deficits, Emotional dysregulation, Perfectionism, Asceticism, and Maturity fears. T scores of 60 and higher were considered elevated. The EDI-C is a version of the measure for use in children 12 years and under. It has 5 subscales: Drive for thinness, Emotional instability, Self-esteem, Overeating and Maturity fears [13]. T scores of 65 and higher were considered elevated.

The EDE-Q is a 33-item self-reported screen used to evaluate for eating disorders for children over 13 years. It measures disordered eating over a 28-day period and is scored across 4 sub-scales: Eating concern, Shape concern, Weight concern, Dietary restraint [14, 15]. It also includes a global score, which is an average of the sub-scales. Subscale scores of 4 and higher were considered elevated.

The ChEAT is a 26-item self-report measure assessing eating behaviors of 9–13 year olds [16]. The three subscales include: Dieting, Bulimia/Food preoccupation and Oral control. Items are scored as a total score, which is a sum of all the item ratings. A total score of 20 and higher was considered elevated.

### Statistical analysis

Analysis comprised of descriptive statistics. Data were collected and stored electronically in a spreadsheet format. Central tendency of continuous measures were represented using means and the variability with standard deviations and the range of each variable. Categorical variables were represented with percentages along with the actual counts so that missing measures are apparent.

Consent to participate was required by all patients who met criteria to be included and were actively receiving eating disorder treatment in the tertiary care program. This study was approved by the Research Ethics Board at SickKids.

## Results

At the time of the study, a total of 369 patients were assessed by the eating disorder program between May 2013 and April 2016; after a comprehensive eating disorder assessment, 31 (8.4%) received a DSM-5 diagnosis of ARFID. The diagnoses of other children and adolescents during that time are outlined in Table 1. Three of the 31 patients with ARFID declined to participate in the study. Therefore, 28 out of the 31 patients diagnosed with ARFID had a complete chart review.

**Table 1:** Diagnosis of patients in a tertiary care pediatric eating disorder program between May 2013 and April 2016

Diagnoses	Number, (%)
ARFID	31, (8.4%)
Anorexia Nervosa	274, (74.3%)
Other Specified Feeding and Eating Disorders	21, (5.7%)
Bulimia Nervosa	17, (4.6%)
Unspecified Feeding and Eating Disorder	5, (1.4%)
Binge Eating Disorder	2, (0.5%)
Diagnosis other than an eating disorder	19, (5.1%)

The diagnosis of ARFID was made using the DSM-5 criteria. The prevalence and breakdown of the categories for the first diagnostic criteria - weight loss or failure to achieve appropriate weight gain; nutritional deficiency; dependence on enteral feeding or nutritional supplements; or significant interference with psychosocial functioning [1] were examined in this cohort. The diagnostic criteria used by clinicians to make a diagnosis of ARFID in this population included weight loss or failure to achieve appropriate weight gain in 96.4% (27/28) and dependence on nutritional supplements in 3.6% (1/28). No children or adolescents were diagnosed due to nutritional deficiency or significant interference with psychosocial functioning.

Table 2 demonstrates the clinical characteristics of patients who received a diagnosis of ARFID. Of the 31 patients with a diagnosis of ARFID, 64.5% (20/31), were female with a mean age of 13.2 (SD = 2. 3; range = 9.3–17.6 years). The duration of illness prior to diagnosis was 28.9 months (SD = 39.6; range = 1–153) and 60.7% (17/28) of patients had a duration of illness of longer than 12 months. Table 3 displays the presenting symptoms of patients with ARFID. All of the patients had 2 or more physical symptoms, such as abdominal pain, vomiting or early satiety. Of interest, almost all the patients had decreasing portion sizes and greater than 50% of the group reported food avoidance, history of nausea, early satiety or abdominal pain. In addition, 71.4% (20/28) of patients reported a trigger for their eating disturbance. These included abdominal pain (*n* = 5), bullying (*n* = 3), death of a family member or friend (*n* = 2), starting a medication (*n* = 2), having emesis (*n* = 2) or witnessing emesis (*n* = 1), concern for food allergy (*n* = 2) and concern for animal rights (*n* = 1).

**Table 2:** Clinical characteristics of children and adolescents with ARFID

Characteristics	Mean ± SD, *n* (Range)
Age (years)	13.2 ± 2.3, 31 (9.3–17.6)
Patients < 12 years old	35.5%, (11/31)
Female patients	64.5%, (20/31)
BMI (kg/m2)	15.8 ± 2.2, 28 (12.2–20.2)
Percent of target goal weight	81.9 ± 8.2%, 28 (65.0–94.6)
Target goal weight < 80%	39.3%, (11/28)
Body weight lost	9.6 ± 9.1%, 28 (0–27.9)
Failure to achieve appropriate weight gain, no weight loss	39.3% (11/28)
Length of illness (months) prior to diagnosis	28.9 ± 39.6, 28 (1–153)
Evaluated for eating disturbance in past	46.4% (13/28)
Heart rate < 50 bpm or SBP < 80 mmHg	7.1% (2/28)

**Table 3:** Presenting symptoms of children and adolescents with ARFID

Presenting symptom	%, *n*
Decreasing portion sizes	96.4, (27/28)
Reported trigger for eating disturbance	71.4, (20/28)
Avoiding specific foods	64.3, (18/28)
History of nausea	60.7, (17/28)
Early satiety	57.1, (17/28)
History of abdominal pain	50, (14/28)
Fear of vomiting	46.4, (13/28)
History of being a picky eater	46.4, (13/28)
History of nutritional supplement use	39.3, (11/28)
Food texture/sensory issues	25, (7/28)
History of fear of chocking	21.4, (6/28)
Fear of contamination of food	21.4, (6/28)
Aversion to liquids	21.4, (6/28)
Count calories	10.7, (3/28)

Almost half of the patients (46.4%, (*n* = 13)), with a diagnosis of ARFID were seen by other specialists or sub-specialists for their eating disturbance prior to being referred to the tertiary care pediatric eating disorder program. These sub-specialists included psychiatry (*n* = 6), endocrinology (*n* = 3), nutrition (*n* = 2), allergy (*n* = 1) and gastroenterology (*n* = 1).

Patients with ARFID were found to present with significant weight loss or other medical compromise. In this study 17.9% (*n* = 5) of patients had lost more than 20% of their body weight before receiving a diagnosis. In addition, 39.3% (*n* = 11) presented with a weight of less than 80% of their TGW. Admission to hospital occurred in in 57.1% (16/28) of patients; 9 were admitted because of a body weight that was less than 80% of the TGW, 6 were admitted for failing to gain weight as an outpatient and 1 was admitted because of bradycardia.

Not all patients who received a diagnosis chose to be followed by our eating disorder program. Over the study period, 32.2% (10/31) of patients who received a diagnosis of ARFID at the initial assessment were either followed by a different eating disorder program (*n* = 6) or a primary care provider (*n* = 4).

**Fig. 1:**
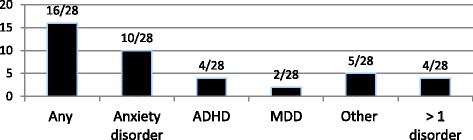
Clinically Diagnosed ^a^ Co-morbid Psychiatric Disorders. ^a^Diagnosed at initial eating disorder assessment by psychiatrist or psychologist through diagnostic interview and review of psychometric measures

Table 4 outlines selected psychometric data for patients with ARFID. The sample size for the psychometric measures was highly variable. Each individual psychometric measure was given to a patient if they were within the age range that the measure was validated for. In addition, some patients may have failed to complete the entire battery of psychometric measures that were given to them. Figure 1 demonstrates the psychiatric comorbidities reported by the psychiatrist or psychologist who assessed the patients with ARFID. A co-morbid psychiatric diagnosis was present in 57.1% (16/28) of patients. Of the patients with a psychiatric co-morbidity, more than half (*n* = 10) had a co-morbid anxiety disorder. In addition, 25% (4/16) of the patients with a psychiatric co-morbidity had more than 1 co-morbid diagnosis.

**Table 4:** Results of psychometric tests in patients with ARFID

Psychometrics test	%, *n*
Elevated total CDI 1 or 2 score	0, (0/24)
Elevated total MASC1 or 2 score	16.7, (4/24)
Elevated total ChEAT score	27.3, (3/11)
Elevated score on any EDE-Q subscale	0, (0/13)
Elevated score on Drive for Thinness, Bulimia, Body Dissatisfaction and ED Risk Composite Subscales on EDI-3	0, (0/11)
Elevated score on Drive for Thinness, Self-Esteem, Overeating and Maturity Fears Subscales on EDI-C	0, (0/10)
Elevated score on Emotional Instability Subscale on EDI-C	20, (2/10)

## Discussion

This is the first study to report the incidence of ARFID in a tertiary care pediatric eating disorder program in a group of children and adolescents that received the DSM-5 diagnosis at the time of their initial assessment. To date, all published studies reporting the incidence of ARFID use assessments which have been retrospectively reclassified using the DSM-5 diagnostic criteria. The incidence was found to be 8.4% and is consistent with previously published literature that used reclassified data from tertiary care eating disorder programs [3, 4].

This study demonstrates that the clinical presentation of children and adolescents with ARFID is complex with multiple physical symptoms. This has been demonstrated in other studies that have applied diagnostic criteria retrospectively [3]. The high number of patients that had a reported trigger for the onset of their eating disturbance highlights that although there is a prolonged duration of illness in many children and adolescents with ARFID, symptoms often have a clearly delineated onset.

Because research on ARFID is limited, clinical understanding of this new diagnosis has depended largely on retrospective chart review and case studies. This study reveals that the majority of children and adolescents presented with decreasing portion sizes and greater than half presented with symptoms such as food avoidance, history of nausea, early satiety or abdominal pain; all non-specific symptoms. The non-specific presentation of ARFID is highlighted by noting that almost half of the patients (*n* = 13) had their eating disturbance assessed in the past by a different sub-specialist. This suggests that in many cases primary care providers and sub-specialists are not recognizing the presentations of these patients as being consistent with an eating disorder and more specifically are not identifying children and adolescents with ARFID. Although these non-specific presenting symptoms on their own may make a diagnosis of ARFID challenging, it is important for future research to understand how to support the diagnosis of challenging cases so that prompt and appropriate referral can occur without lengthening the duration of illness. The long interval between developing a nonspecific cadre of symptoms, being diagnosed with an eating disorder and starting treatment has the potential to result in medical complications and effect on quality of life. Earlier recognition of ARFID and implementation of eating disorder treatment may prevent medical compromise and hospitalization in some patients. Further, it may also inform broader public health prevention strategies.

There is an inherent selection bias in this sample. The patients in this study are from a pediatric tertiary care eating disorder center (8–18 years old). The majority of the referrals include patients who have a significant weight loss, growth failure or were medically unstable [7]. As such, this study population is less likely to include patients who present with no weight loss, patients who are younger than 8 years old and patients who would receive an ARFID diagnosis because of a significant interference with psychosocial functioning. Thus, this study sample may not be representative of the larger pediatric ARFID population.

Little prior data exists on the psychological characteristics and psychometric results of children and adolescents receiving a diagnosis of ARFID. In this study, none of the patients had elevated scores on the CDI. This suggests that depression, based on the psychometric measures, was not present in children and adolescents with ARFID. This finding is consistent with Nicely et al. who showed that patients who retrospectively met criteria for ARFID had a lower comorbidity of depression than those who met criteria for other eating disorders [5]. Two patients however, had a clinical diagnosis of major depressive disorder (2/28) but did not have elevated scores on the CDI. The CDI is often used as a screen for depression and should not be used to diagnose depression, but rather corroborate the clinical diagnosis [17]. Therefore, one needs to use caution when interpreting the results from self-report inventories like the CDI. Finally, this study included a relatively small sample size and consequently the results need to be carefully interpreted in this context.

This study found that only 16.7% (4/24) of patients had elevated scores on the MASC. This is fewer than expected based on the number of patients in the study with clinically diagnosed anxiety disorders (10/28) at the time of assessment. Nicely et al. found that Revised Children’s Manifest Anxiety Scale scores were not different between patients who met criteria for ARFID and patients who met criteria for AN. However, they found that patients who met criteria for ARFID had significantly more clinically diagnosed anxiety disorders than those with AN. This highlights a few interesting considerations. First, the discrepancies between the scores on self-report measures of anxiety and the clinical diagnosis of anxiety may be accounted for in part by difficulty in responding to the questions (e.g., inability to understand questions). Further, these instrument may not be suitable to discriminate between psychometric and clinical diagnosis in tertiary care populations of children with ARFID. Finally, complexity is introduced by the fact that anxiety disorders differ in type and symptoms, coupled with recent evidence that suggests that ARFID includes a variety of subtypes. Thus, the current psychometric measure may not be able to assess the complexities of anxiety in the context of a heterogeneous diagnosis like ARFID. Further research is needed in developing and evaluating psychometric measures for anxiety in children and adolescents with ARFID.

Nicely et al. also found that patients who retrospectively met criteria for ARFID had significantly lower total scores on the ChEAT than patients with other eating disorders, indicating fewer classic eating disorder behaviors [5]. We found that 3/11 patients had elevated scores on the ChEAT. All patients who had elevated scores were highest on the oral control scale. Nicely et al. found that there was no significant difference between patients who met criteria for ARFID and patients who met criteria for other eating disorders on the oral control subscale [5]. Dovey et al. assessed screening for ARFID using the Behavioral Pediatrics Feeding Assessment Scale and the Child Food Neophobia Scale and found that these measures were both able to discriminate clinical and non-clinical cases of ARFID in children ages 24 to 84 months [18]. At present, there are no reliable and validated psychometric tests used to assess for ARFID in older children and adolescents.

The EDE-Q is one of the most widely used eating disorder assessments [19], however we found that none of the 13 patients who completed this measure had elevated scores on any EDE-Q subscale. In addition, none of the patients had elevated scores on Drive for Thinness, Bulimia, Body Dissatisfaction and ED Risk Composite Subscales on EDI-3 or on the Drive for Thinness and Overeating subscales on the EDI-C. Our study suggests that patients with ARFID may not be identified on psychometric screens commonly used to evaluate eating disorders such as AN or BN. Future research should consider modifying existing tools or developing new psychometric tools to help in the evaluation of children and adolescents with ARFID, as currently used psychometric scales are not useful in identifying young people with this diagnosis.

Limitations to this study include its retrospective design and limited sample size. Despite these limitations this study provides insight into the often complex clinical presentations of patients with ARFID. The non-specific nature of ARFID frequently results in delayed diagnosis even in those with growth failure and medical compromise. Future retrospective and prospective studies that further characterize ARFID and examine treatments are needed.

## Conclusion

This is the first study to retrospectively determine the incidence of ARFID in children and adolescents diagnosed with the DSM-5 diagnostic criteria at their initial assessment. This study confirms that there is a very heterogeneous group that is receiving the diagnosis within an eating disorder treatment program. The clinical presentation of children and adolescents with ARFID is complex with multiple physical symptoms and comorbid psychiatric disorders. Commonly used pediatric eating disorder psychometric scales are not useful in identifying children and adolescents with ARFID. As we learn more about the diagnostic category of ARFID we recognize that this is a heterogeneous group of patients. Perhaps a greater emphasis on understanding ARFID subtypes will help us to better delineate the characteristics and symptoms that will aid in developing specific psychometric scales. Research on the prevalence and psychopathology of ARFID in the pediatric population is still in its infancy and is limited by the lack of validated instruments to measure these eating behaviors and associated comorbidities. This study incorporated a standard battery of psychometric tests that has historically been used in our tertiary care pediatric eating disorder program even before the advent of ARFID as a diagnostic category in the DSM-5. This study identified the limitations of these psychometric tests in this population. Clearly, future research on ARFID should include the development of valid and reliable psychometric tests that focus on assisting both in the diagnosis of ARFID and its associated mental health disorders. Psychometric properties that tap into the severity of ARFID, the complexity of the diagnostic category, and the degree to which ARFID is impairing the child’s ability to function will further assist in formulating a comprehensive treatment.

## Abbreviations

AN: Anorexia nervosa
ARFID: Avoidant/Restrictive Food Intake Disorder
BMI: Body mass index
BN: Bulimia nervosa
CDI: Children’s Depression Inventory
ChEAT: The Children’s Eating Attitudes Test
DSM-5: *Diagnostic and Statistical Manual of Mental Disorders, 5th edition*
EDE- Q: Eating Disorder Examination Questionnaire
EDI: Eating Disorder Inventory
MASC: Multidimensional Anxiety Scale for Children
TGW: Target goal weight

## Footnotes

1 diagnosed at comprehensive eating disorder assessment by psychiatrist or psychologist through diagnostic interview and review of psychometric measures.
